# The potential role of the gut microbiota in the development of autoantibodies associated with Spondyloarthritis: a narrative review

**DOI:** 10.1186/s12865-026-00855-3

**Published:** 2026-06-15

**Authors:** Alaa Elsaghir, Al-Hassan Soliman Wadan, Torsten Witte

**Affiliations:** 1 Department of Rheumatology and Immunology, Hannover Medical School, Hannover, 30625 Germany; 2 Department of Microbiology & Immunology, Faculty of Pharmacy, Assiut University, Assiut, 71515 Egypt; 3 Oral Biology Department, Faculty of Dentistry, Galala University (15888), Galala Plateau, Attaka, Suez, Suez Governorate Egypt

**Keywords:** Spondyloarthritis, Gut dysbiosis, Autoantibodies, Microbiota, Gut-immune axis, Microbial Therapy

## Abstract

Spondyloarthritis (SpA) is a set of immune-inflammatory conditions characterized by musculoskeletal and extra-articular manifestations. Increasing evidence indicates that alterations in the gut microbiota (dysbiosis) may influence both mucosal and systemic immune responses, potentially contributing to the loss of tolerance and the development of autoantibodies in SpA.

This narrative review examines the current evidence linking gut dysbiosis to autoantibody development in SpA, with particular focus on ankylosing spondylitis (AS) and psoriatic arthritis (PsA).

We summarized key mechanistic pathways, including Th17 axis activation, molecular mimicry, increased intestinal permeability (“leaky gut”), and altered microbial metabolite signaling. We discussed the potential relevance of these mechanisms to SpA-associated autoantibodies such as anti-CD74, anti-HSP65, and anti-Kaiso. Where direct evidence in SpA is limited, findings from other autoimmune diseases are considered as mechanistic analogies rather than definitive parallels.

We further review microbiome-targeted therapeutic strategies, including probiotics, prebiotics, and bacterially based therapies, and highlight differences between preclinical findings and available clinical data. Although biologically plausible mechanisms, direct causal evidence linking gut dysbiosis to autoantibody production in SpA remains limited, and clear methodological heterogeneity persists across microbiome studies.

Overall, while modulation of the gut-immune axis represents a promising research direction in SpA, further mechanistic and longitudinal human studies are required before microbiota-targeted interventions can be considered applicable for autoantibody modulation.

**Graphical Abstract:**

**Figure:**
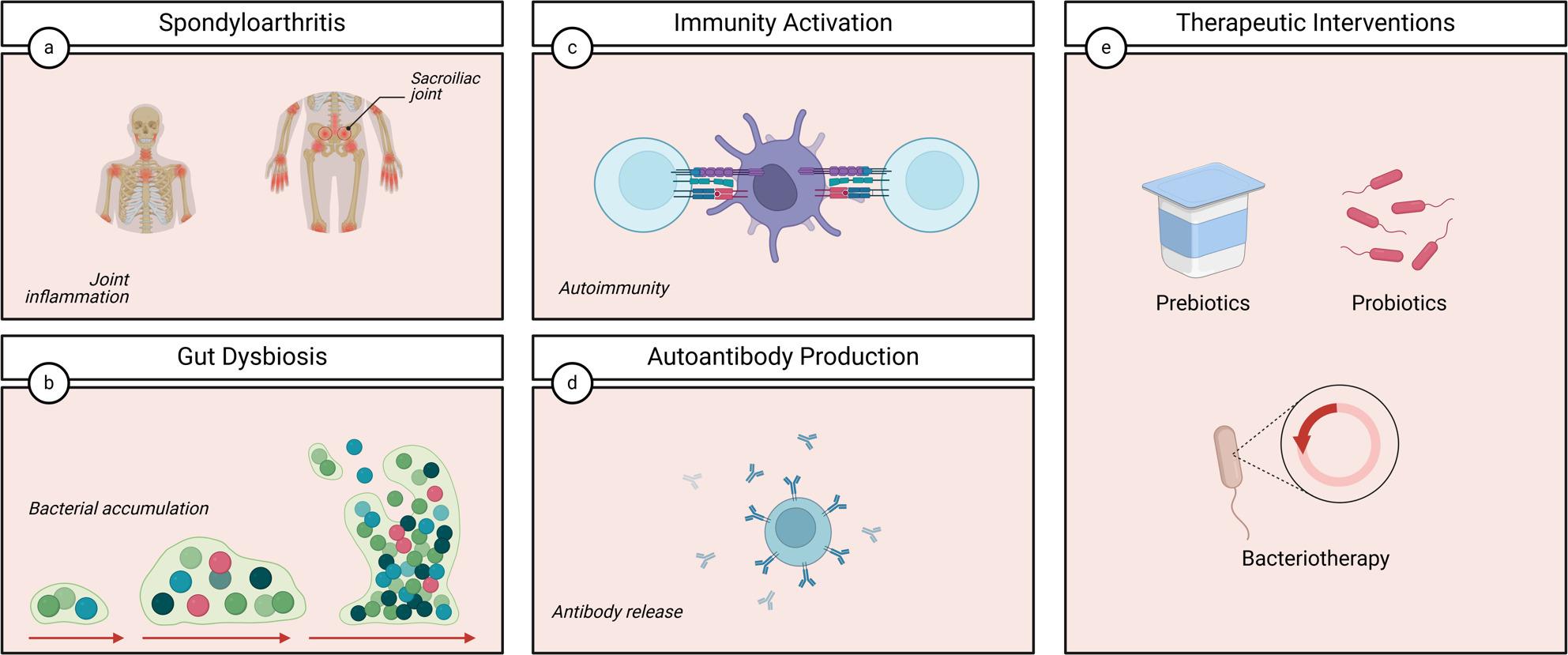
The graphical abstract illustrates the role of gut microbiota in SpA (a), highlighting how gut dysbiosis (b) contributes to immune dysregulation and the development of autoantibodies such as anti-CD74, anti-HSP65, and anti-Kaiso (c,d). Dysbiosis may influence immune responses through mechanisms including T-cell activation, molecular mimicry, and increased intestinal permeability, ultimately contributing to systemic inflammation. The graphical abstract also highlights the association with HLA-B27 and illustrates how microbial imbalance may contribute to axial and peripheral manifestations of SpA. Furthermore, it presents therapeutic approaches targeting gut dysbiosis, including probiotics, bacteriotherapy, and prebiotics (e), while highlighting current challenges and limitations in microbiome research and therapy development. Understanding the gut–immune axis is important for advancing future treatment strategies in SpA.

## Introduction

Spondyloarthritis (SpA) is a diverse set of inflammatory musculoskeletal illnesses characterized by shared clinical characteristics, genetic predispositions, and pathophysiological processes [1–3]. It includes several conditions that are interconnected, such as ankylosing spondylitis (AS), psoriatic arthritis (PsA), reactive arthritis (ReA), SpA associated with inflammatory bowel disease, and other SpA types that are not clearly defined [4]. These conditions share several common clinical features, including enthesitis, dactylitis, peripheral arthritis, and inflammatory back pain. Additionally, there are extra-articular abnormalities such as uveitis, psoriasis, and inflammatory bowel disease (IBD) [2, 5].

SpA mainly presents with sacroiliitis, named as axial SpA (axSpA), and may affect the peripheral joints, namely peripheral SpA (pSpA) [4, 6, 7]. The radiological findings from the sacroiliac joints (SIJ) allow for the further classification of axSpA into two groups: r-axSpA, which is defined as having radiologically confirmed imaging damage, and nr-axSpA, which is defined as non-radiographic axSpA [8–11]. The progression of nr-axSpA to r-axSpA may occur in certain individuals over time [12] Most research on axSpA has focused on r-axSpA, also known as AS [8, 13].

SpA diagnosis and classification are mostly based on radiographic findings of the SIJ or detection of the HLA-B27 gene [14]. Despite magnetic resonance imaging (MRI) being able to identify SpA patients and detect inflammatory alterations of the SIJ in their early stages [15], a significant delay of 5 to 10 years persisted between symptom onset and diagnosis [15]. Interestingly, the MHC class I protein HLA-B27 is linked to all of these conditions to varying degrees [4]. However, having the HLA-B27 gene is not sufficient to cause AS, even though over 90% of AS patients carry it [16]. In addition, although HLA-B27 has a considerable degree of specificity and sensitivity, the percentage of healthy people who test positive for it is as high as 10% [13]. This suggests that additional immunological and environmental factors contribute to disease development.

Unlike conventional autoimmune diseases, SpA has previously been linked to autoantibodies. Emerging evidence suggests that humoral immune responses may be more significant than previously assumed, as some autoantibodies have been detected before the onset of overt clinical or radiographic signs [17]. This discovery requires an in-depth exploration of the processes behind the breakdown of immunological tolerance in SpA.

Increasing interest has been directed toward the gut microbiota as a possible contributor to immunological dysregulation in SpA. The gut microbiota refers to the assemblage of microorganisms residing in the gastrointestinal system, whereas the gut microbiome refers to their collective genetic material and functional capabilities [18]. Experimental research, including that using the K/BxN mouse model of arthritis, has revealed that segmented filamentous bacteria (SFB) residing on the gut mucosa might potentially induce autoimmune arthritis by triggering gut T helper 17 (Th17) cells, leading to increased generation of autoantibodies [19], thereby linking intestinal microbial composition to systemic autoimmunity. Moreover, clinical research has shown a significant incidence of gastrointestinal involvement in SpA, including IBD [20], irritable bowel syndrome (IBS) [21], and microscopic gut inflammation [22]. Therefore, supporting the concept of a gut–joint axis [23].

Collectively, these results indicate that gut microbiota may influence immunological responses relevant to SpA through multiple mechanisms, including Th17 activation, molecular mimicry, and barrier dysfunction (see Sect. "Mechanisms underlying the gut microbiota-immune axis"). These pathways may contribute to loss of immunological tolerance and the emergence of autoantibody responses (see Sect. "Autoantibodies in SpA: Clinical Evidence and Mechanistic Links").

This review critically examines the role of gut microbiota in the generation of autoantibodies in SpA, focusing on underlying immunological mechanisms, emerging clinical data, and potential implications for diagnosis and treatment.

## Search strategy

A structured literature search was conducted using PubMed and Google Scholar was used to select the studies. The main search terms were "spondyloarthritis," "ankylosing spondylitis," "normal microbiota," "gut microbiota," "dysbiosis," "autoantibodies," "links between microbiota and SpA," and "how gut microbiota works in SpA."

The most recent peer-reviewed papers published mostly between 2000 and 2025 were given the highest priority. We focused on human studies, but we also included relevant mechanistic insights from in vitro and animal studies.

The goal of this narrative review is to assemble available information into a clear, organized framework relevant to SpA. Still, it does not follow the usual approach to conducting a systematic review.

## Mechanisms underlying the gut microbiota-immune axis

Understanding the interactions between intestinal microbial populations and the immune system is crucial for elucidating the postulated gut-joint axis in SpA. Gut microbiota execute metabolic, structural, and immunological functions that facilitate mucosal homeostasis and systemic immune modulation. In healthy populations, the gut microbiota is mainly composed of *the Bacteroidetes and Firmicutes phyla, as well as Actinobacteria*, *Proteobacteria*, and other taxa [24]. These microbes interact with host epithelial and immune cells through microbial metabolites, cell-surface molecules, and immune signaling pathways. The gut microbiota contributes to immunological maturation, tolerance induction, and protection against pathogens, giving rise to its description as a functioning "microbial organ “ [19, 25–29].

Although bacteria are the most thoroughly investigated microorganisms of the intestinal ecosystem, other microorganisms, such as viruses, archaea, and fungi, are increasingly recognized as significant contributors to mucosal immunity. Their functions are not fully defined; however, studies indicate that the intestinal microbiome and virome may affect immunological signaling and microbial stability, especially in chronic inflammatory disorders [24, 30–33].

Increasing evidence indicates that changes in microbial composition, known as gut dysbiosis, may influence immunological pathways associated with inflammatory conditions, including SpA [34, 35]. However, the nature of these interactions is still poorly understood. In some cases, microbial changes may indicate a consequence of inflammation, dietary habits, or medication exposure rather than a major causal factor. Consequently, existing data mostly support biological plausibility and correlation, rather than conclusive causation [36, 37].

Various immunological mechanisms have been described to explain how microbial dysbiosis may affect systemic immune activation. These factors encompass activation of the Th17 axis [25, 38–42], molecular mimicry [34], intestinal barrier dysfunction [43], and modifications in microbial metabolites [34]. Each of these mechanisms could contribute to the destruction of immunological tolerance and, perhaps, promote the emergence of autoantibody reactions, as discussed subsequently in this article (see Sect. " Autoantibodies in SpA: Clinical Evidence and Mechanistic Links" for autoantibody evidence).

### Microbiota-driven Th17 axis activation

A commonly proposed mechanism linking intestinal microbiota to inflammatory disorders is the activation of the IL-23/IL-17 immune system, which plays a vital role in the development of SpA. Th17 cells are a subset of CD4^+^ T lymphocytes that produce interleukin-17 and play a role in protecting the host from infections from outside the body. Increased Th17 activation has been linked to numerous immune-mediated inflammatory disorders, including SpA [25, 44–50].

Experimental studies have revealed that the gut microbiota composition significantly affects the development of Th17 cells. SFB can directly colonize intestinal epithelial cells and help Th17 cell proliferation in the lamina propria [51–53]. Germ-free animal models exhibit markedly reduced numbers of IL-17-producing cells, underscoring the role of commensal microbes in shaping Th17 responses [25].

A substantial portion of the molecular data linking microbiota to Th17 activation derives from animal models of autoimmune disease, in which microbial colonization may affect disease susceptibility. SFB colonization has been shown to induce inflammatory arthritis in some mouse models [54]. These findings provide a conceptual framework indicating that microbiota-induced immune activation may affect autoimmune mechanisms.

However, it is crucial to acknowledge that these data predominantly originate from murine models, and direct validation in human SpA is still constrained. Clinical studies have demonstrated increased activation of the IL-23/IL-17 pathway in SpA; however, the specific role of different bacterial species remains unclear [55, 56].

### Molecular mimicry and loss of immune tolerance

Another proposed mechanism by which microbial exposure may influence autoimmune diseases is molecular mimicry, in which microbial antigens resemble host proteins. These similarities may elicit cross-reactive immunological responses, leading to the activation of autoreactive T or B cells [34]. This tendency has been extensively documented in several autoimmune conditions. Microbial peptides analogous to the Ro60 autoantigen have been observed in SLE, suggesting that cross-reactive immune responses might precede the onset of clinical illness [34]. Comparable pathways have been described in multiple sclerosis (MS), where microbial peptides may stimulate T cells that recognize myelin antigens [57–61].

Within the context of SpA, the evidence for molecular mimicry is still limited but biologically plausible. Specific bacterial species linked to ReA, such as *Salmonella, Klebsiella,* and *Shigella,* are suggested to have antigenic similarities with host proteins provided by HLA-B27, a major genetic risk factor for SpA. Such interactions may hypothetically induce immune responses that exceed the host's capacity to respond to microbial antigens, leading to chronic inflammation [62, 63]. Nevertheless, direct evidence of molecular mimicry resulting in autoantibody production in SpA remains absent. The current data consequently endorse this process as a hypothesis necessitating additional experimental validation, highlighting a significant knowledge gap in the field.

### Intestinal barrier dysfunction (“leaky gut”)

The intestinal epithelium acts as a vital barrier that isolates the luminal microbiota from host immunological tissues. Disruption of this barrier can lead to increased intestinal permeability, commonly known as "leaky gut," and may allow microbial products, such as lipopolysaccharide (LPS) and other bacterial constituents, to enter the systemic circulation and trigger immunological responses [43].

Several studies have shown that intestinal barrier disruption may occur in patients with AS. Higher levels of zonulin, a modulator of epithelial tight junctions, have also been observed in ileal tissue and blood samples from AS patients, indicating compromised barrier integrity [43, 64]. Moreover, irregularities in tight junction proteins, including Occludin and Claudin, have been documented in intestinal tissue from patients with SpA [65].

Animal studies provide more support for this hypothesis. In HLA-B27 transgenic rats, alterations in gut microbial composition are associated with changes in epithelial barrier function and intestinal inflammation [66–69]. Nonetheless, extrapolating these findings to human studies is complex because medications, dietary components, and systemic inflammation can influence intestinal permeability [70–72].

Notwithstanding these concerns, barrier failure is widely recognized as a potential factor in the gut–joint axis, as heightened exposure to microbial antigens may enhance systemic immune activation and promote the emergence of autoreactive immune responses [72–74].

### Microbial metabolites and immune regulation

In addition to direct microbial interactions with immune cells, gut bacteria affect host immunity by producing bioactive metabolites. Short-chain fatty acids (SCFAs), such as acetate, propionate, and butyrate, are among the most thoroughly studied compounds produced during the fermentation of dietary fiber [34, 75–77].

SCFAs exert several immunomodulatory effects. They facilitate the proliferation of regulatory T cells (Tregs), augment epithelial barrier integrity, and inhibit excessive inflammatory responses at mucosal surfaces. Lower levels of SCFA-producing bacteria have been documented in various inflammatory disorders, suggesting that altered microbial metabolism may contribute to immunological dysregulation [76, 78–86].

Dietary habits significantly affect the synthesis of these metabolites by altering gut microbiota composition [83, 87, 88]. Thus, disruptions in microbial metabolic activity, rather than the presence or absence of specific bacterial species, may be a significant factor in immunological homeostasis [84–86, 89].

In the context of SpA, modifications in microbial metabolic pathways have been documented in numerous investigations; however, the therapeutic implications of these results are still being examined [90, 91].

Collectively, the above mechanisms illustrate different ways in which modifications in gut microbiota may affect immune modulation. Dysbiosis may facilitate Th17-mediated inflammation, compromise epithelial barrier integrity, increase exposure to microbial antigens, and modify the synthesis of immunoregulatory metabolites. These activities may disrupt immunological tolerance and, in turn, promote the emergence of autoantibody responses. Although direct causal relationships are not fully established, these mechanisms offer a conceptual framework for understanding the disease-specific observations detailed in the subsequent section, which more comprehensively analyzes current evidence on gut dysbiosis in SpA and associated autoimmune disorders.

## Gut dysbiosis and autoimmune disorders in spondyloarthritis

Gut dysbiosis occurs due to alterations in the diversity, composition, or functionality of the intestinal microbiota [92] Gut flora imbalances are strongly associated with SpA [93]. More than sixty percent of patients with AS show subclinical intestinal inflammation [22], with an additional 4–16% progressing to clinically apparent IBD [94] supporting a gut-joint axis. Moreover, pathogens such as *Yersinia, Shigella, Campylobacter*, and *Salmonella* are linked to ReA [95], while *Klebsiella pneumoniae* has been implicated in AS. [96]

**Fig. 1:**
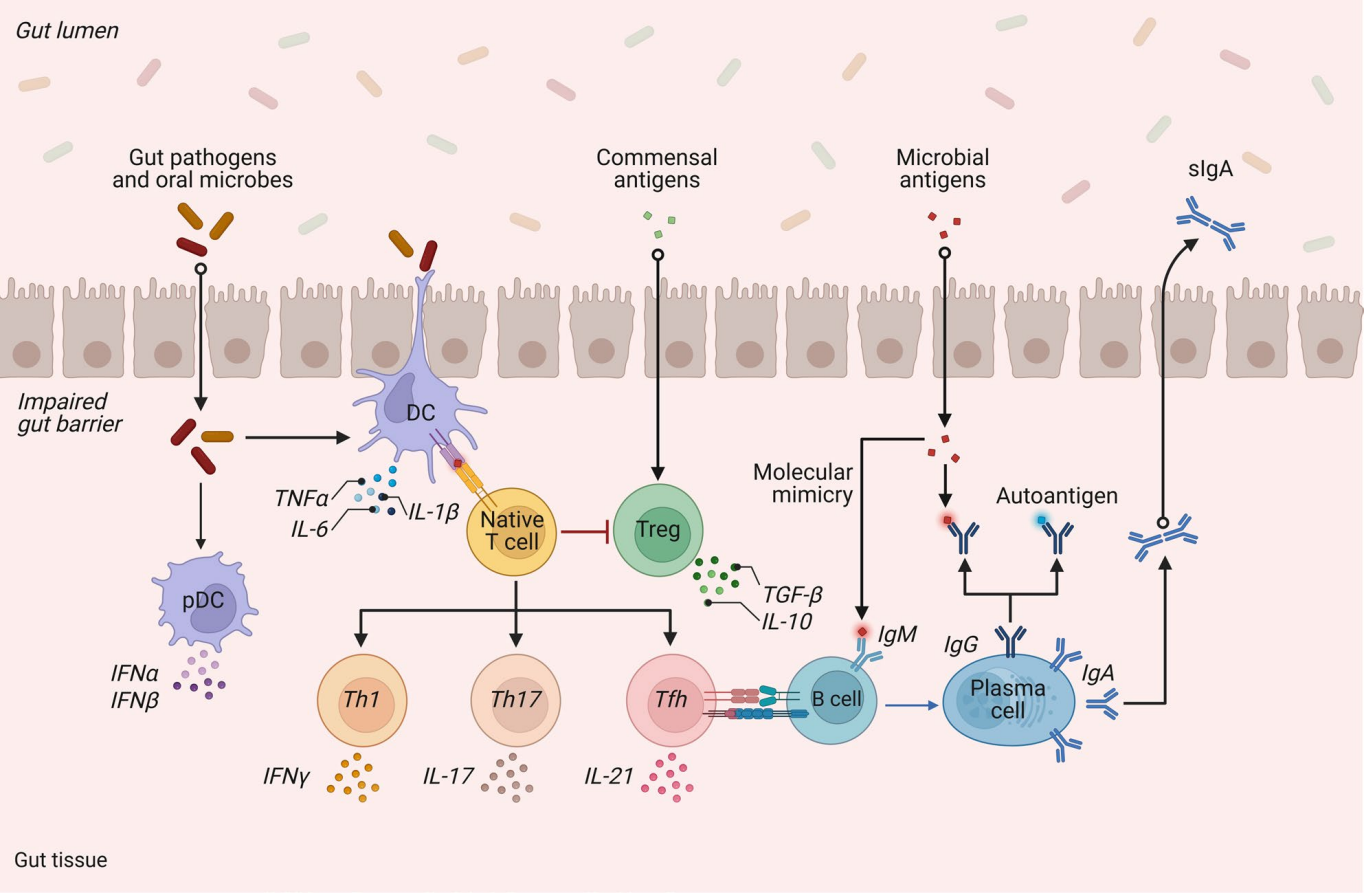
Mechanisms linking gut microbiota dysbiosis to autoimmune responses. Gut microbiota imbalance (dysbiosis) may contribute to immune dysregulation through multiple interconnected pathways. Disruption of intestinal barrier integrity increases permeability, facilitating translocation of microbial antigens across the epithelium. These antigens are internalized by antigen-presenting cells (APCs), including dendritic cells (DCs) and macrophages, thereby activating innate and adaptive immune responses. Innate immune activation includes the stimulation of plasmacytoid dendritic cells (pDCs) and the production of pro-inflammatory cytokines, such as type I interferons. In parallel, microbial antigens promote activation of CD4+ T cells and their differentiation into pro-inflammatory subsets, including T helper 1 (Th1), T helper 17 (Th17), and T follicular helper (Tfh) cells. These responses support B-cell activation and differentiation into plasma cells, resulting in the production of immunoglobulin A (IgA) and autoantibodies. Additionally, dysbiosis is associated with reduced production of immunoregulatory microbial metabolites, such as short-chain fatty acids (SCFAs), which impairs regulatory T cell (Treg) function and further promotes inflammation. Molecular mimicry between microbial and self-antigens may also contribute to the loss of immune tolerance and the generation of autoantibodies [97, 98]. Abbreviations: APCs, antigen-presenting cells; DCs, dendritic cells; pDCs, plasmacytoid dendritic cells; Th, T helper cells; Tfh, T follicular helper cells; Treg, regulatory T cells; SCFAs, short-chain fatty acids

Dysbiosis increased intestinal permeability, facilitating microbial translocation and activating both innate and adaptive immunity. This boosts Th1/Th17 responses, lowers Treg activity (partly by reducing short-chain fatty acids), and may cause autoimmunity via molecular mimicry [97, 98], as illustrated in Fig. 1.

### Gut microbiota and SpA

#### I-axSpA/AS

**Fig. 2:**
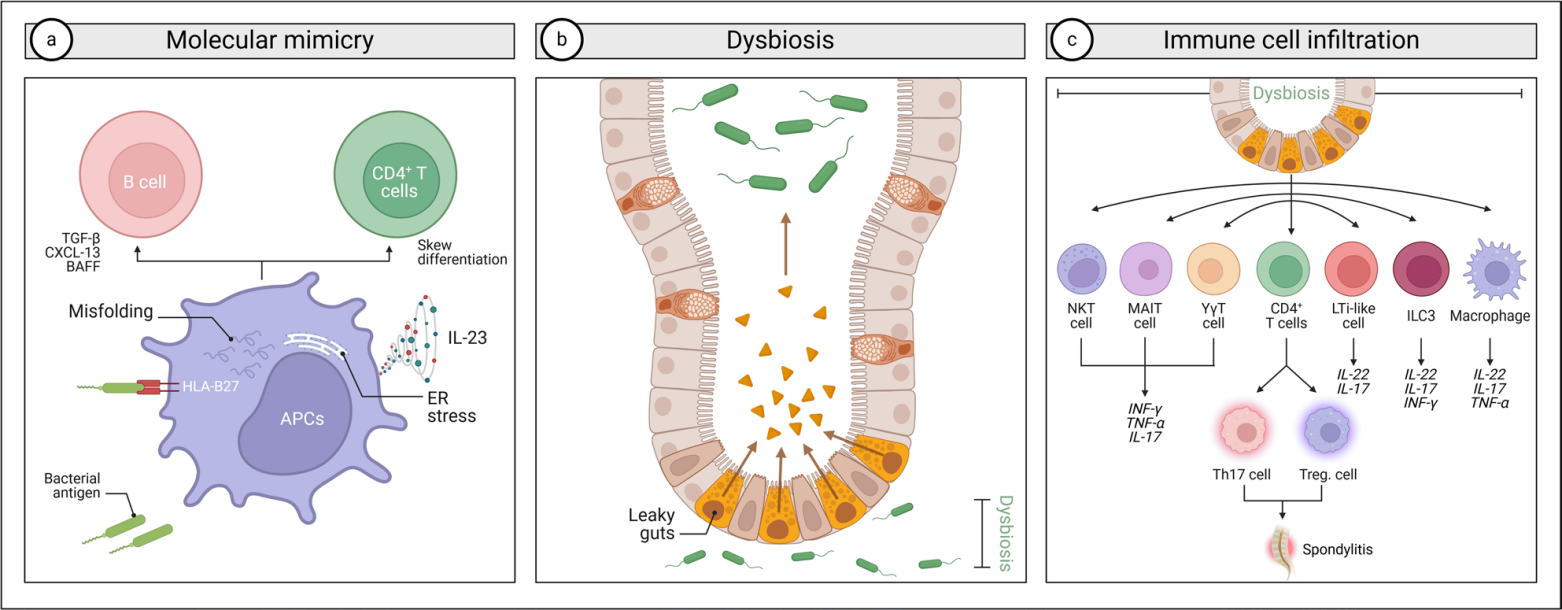
Proposed mechanisms linking gut microbiota, HLA-B27, and immune dysregulation in ankylosing spondylitis. **a** HLA-B27–microbiota interactions: HLA-B27 may present microbial-derived peptides (e.g., from *Klebsiella* and *Shigella*) to antigen-presenting cells (APCs), potentially leading to cross-reactive immune responses through molecular mimicry. In addition, misfolding of HLA-B27 within the endoplasmic reticulum (ER) may induce ER stress and activate the unfolded protein response (UPR) or autophagy pathways, promoting inflammatory signaling and activation of the IL-23/IL-17 axis. These processes may contribute to B-cell activation and increased IgA production, although direct evidence in human SpA remains limited. **b** Intestinal barrier dysfunction: Dysbiosis may disrupt tight junction proteins (e.g., Claudins), reduce mucus layer integrity, and impair the gut barrier, facilitating the translocation of microbial products into the systemic circulation. The extent and clinical relevance of bacterial translocation in ankylosing spondylitis remain incompletely defined. **c** Immune cell activation and cytokine signaling: Dysbiosis-driven immune activation within the gut mucosa involves multiple immune cell populations, including type 3 innate lymphoid cells (ILC3), mucosal-associated invariant T (MAIT) cells, Th17 cells, regulatory T (Treg) cells, natural killer T (NKT) cells, γδ T cells, macrophages, and dendritic cells. These cells produce pro-inflammatory cytokines, including IL-17, IL-23, TNF-α, IFN-γ, and IL-22, which contribute to systemic inflammation and joint involvement. Dysregulation of the Th17/Treg balance is a key feature in ankylosing spondylitis pathogenesis [100]. Abbreviations: APCs, antigen-presenting cells; ER, endoplasmic reticulum; UPR, unfolded protein response; ILC3, type 3 innate lymphoid cells; MAIT, mucosal-associated invariant T cells; Treg, regulatory T cells

The presence of gut microbial imbalances is increasingly recognized as a shared feature of several inflammatory conditions, particularly SpA [18]. SpA also shares several clinical and pathogenic characteristics with IBD, a condition strongly linked to alterations in the gut microbiota [99], as shown in Fig. 2.

In animal models, rats with excessive HLA-B27 genes develop spontaneous inflammatory disease characterized by arthritis and colitis, which is similar to human SpA [101]. Rosenbaum et al. hypothesized that HLA-B27 may alter the composition of the intestinal microbiota, increasing susceptibility to specific diseases linked to this genetic variant [95]. Disruption of the gut environment, including increased intestinal permeability, may induce microbial imbalance and epithelial inflammation, contributing to systemic immune dysregulation. [18].

The presence of beneficial gut microbes is necessary for normal immune system development. Animals reared in germ-free environments do not develop functional lymphoid organs and exhibit compromised adaptive immune responses [102]. HLA-B27 transgenic rats reared in germ-free conditions consistently do not exhibit SpA-associated inflammation, including colitis or arthritis; however, these symptoms reappear upon the introduction of normal flora [103, 104]. Several murine studies have shown that both the overall microbial population and specific species significantly influence the onset of inflammation-related arthritis [18, 105].

In human studies, AS patients exhibit unique microbial colonization in the terminal ileum. *Ruminococcaceae*, *Prevotellaceae*, and *Lachnospiraceae* are more abundant in AS patients, whereas Actinomyces and Streptococcus are reduced compared to healthy controls [106–108]. Functional studies show decreased IL-10 production when peripheral blood mononuclear cells from AS patients are stimulated with autologous Bacteroides [109].

Administration of prebiotics in HLA-B27 transgenic rats with SpA improves colitis, highlighting the therapeutic potential of targeting the gut microbiota [18, 110]. Additionally, HLA-B27 expression in human monocytic cells reduces their ability to handle *Salmonella* [111] and to proliferate in response to lipopolysaccharides (LPS), indicating that HLA-B27 shapes the gut microbiota through intracellular effects [112].

While NOD2 gene variants were common in SpA patients with chronic gut inflammation, their frequency does not differ in SpA individuals with acute gut inflammation or without gut inflammation compared to the healthy population [112]. Collectively, these findings support the hypothesis that alteration in gut microbial composition may contribute to immune dysregulation and inflammation in axSpA.

#### II-juvenile spondyloarthritis

A subset of juvenile SpA, known as enthesitis-related arthritis (ERA), shows a microbial pattern similar to that of adult AS patients [108]. ERA patients demonstrate reduced abundance of Clostridium leptum [112] and lower abundance of *Fecalibacterium prausnitzii* compared to healthy subjects. Despite these microbial differences, levels of blood IgG and IgA antibodies against *B. fragilis and F. prausnitzii* are comparable between patients and controls.

Juvenile SpA patients also exhibit a cellular immune response to the outer membrane proteins of *Salmonella typhimurium*, which is not observed in healthy individuals [113]. Analyzing the microbiota of young SpA individuals revealed two distinct groups: one dominated by Bacteroides and the other by *Akkermansia muciniphila*.

These results indicate that alterations in gut microbiota may lead to immunological dysregulation in juvenile SpA and suggest molecular similarities with adult SpA, therefore highlighting the importance of gut microbiota in spondyloarthropathies [18].

### Evidence in PsA

A considerable proportion of individuals with psoriasis (Pso) and psoriatic arthritis (PsA) exhibit subclinical gastrointestinal inflammation, indicating a possible association between intestinal immune activity and joint disease [114].

Several studies have reported reduced microbial diversity in PsA patients, primarily due to decreased abundances of multiple bacterial taxa [115]. In particular, a negative correlation between *Coprococcus* abundance and psoriasis has been observed, regardless of arthritis status. In contrast, reduced abundance of *Akkermansia* and *Ruminococcus* appears to be more specifically associated with psoriasis accompanied by arthritis. Interestingly, *Ruminococcus* reduction has also been observed in IBD, suggesting potential shared microbial characteristics among immune-mediated inflammatory disorders [116]. Further research has revealed a diminished prevalence of *Alistipes* in both PsA and Crohn’s disease, indicating broader alterations in microbial populations associated with mucosal homeostasis [115, 116].

Many of these microorganisms are involved in mucus degradation and SCFAs production, processes that are important for maintaining intestinal homeostasis and immune regulation. A reduction in these commensal bacteria may therefore disrupt microbial balance and contribute to immune dysregulation in PsA [18]. Collectively, these data support the hypothesis that alterations in gut microbiota may contribute to the inflammatory mechanisms driving PsA.

### Gut microbiota and rheumatoid arthritis (RA)

RA is often employed as a comparative model to investigate the impact of gut microbiota alterations on autoimmune diseases. Several studies conducted in Europe, North America, and Asia report that individuals with RA exhibit distinct gut microbial profiles compared with healthy controls, suggesting a possible association between microbial imbalance and immune dysregulation [34]. Observational studies have reported an increased prevalence of *Prevotella copri* in newly diagnosed RA patients, together with a reduced prevalence of microbial groups such as *Bifidobacteria, Bacteroides*, *Eubacterium rectale*, *Clostridium coccoides*, and the *Bacteroides fragilis* subgroup [117–119]. Some studies have proposed that *P. copri* proliferation may facilitate disease development, particularly in people with reduced genetic predisposition, in whom environmental factors may exert a greater influence [120]. However, other studies have failed to validate this connection, underscoring the diversity within cohorts and indicating that the directionality of microbiome alterations remains ambiguous [121]. Other species, such as *Collinsella, Eggerthella,* and *Faecalibacterium*, have been identified in fecal samples from those with RA. Elevated levels of *L. iners, L. ruminis,* and *L. salivarius* were noted in healthy individuals [122, 123].

Evidence from animal models indicates that variations in microbiota may affect the progression of arthritis. In a collagen-induced arthritis (CIA) mouse model, Rogier and colleagues observed an increased prevalence of bacterial families, including *Lachnospiraceae, Desulfovibrionaceae,* and *Ruminococcaceae*, during the immune-priming phase of arthritis, alongside a decrease in *Bacteroidaceae* [124]. Experimental studies have shown that antibiotic treatment can reduce arthritis severity in mice, partly by depleting segmented filamentous bacteria, which are known to enhance mucosal immune activation and the development of Th17 cells [124, 125]. These mechanistic discoveries, primarily derived from mouse models, offer a conceptual framework indicating that microbiota-driven immune regulation may play a role in systemic autoimmunity. Collectively, these data reveal the potential impact of microbial dysbiosis on immunological pathways associated with autoimmune arthritis. Although these discoveries mostly stem from RA research, they offer mechanistic insights that may also enhance studies examining gut microbiota-immune interactions in SpA.

## Autoantibodies in SpA: clinical evidence and mechanistic links

Rheumatic illnesses include a diverse array of diseases marked by systemic symptoms, immunological irregularities, and the production of autoantibodies. These autoantibodies function as prospective biomarkers for diagnosis, disease categorization, activity assessment, and prognosis [126].

Unlike RA, which includes well-known serological markers such as anti-citrullinated protein antibodies and rheumatoid factor, SpA has historically been classified as "seronegative." However, growing evidence indicates the presence of specific autoantibodies in certain patient cohorts, particularly in axSpA and PsA [127].

Currently, there is no single autoantibody for SpA; however, several potential candidates exist that may enhance diagnosis, elucidate the disease's etiology, or monitor its progression. Several mechanisms may contribute to the pathophysiology of SpA autoantibodies, including immunological activation, dysbiosis of the gut microbiota, and abnormal bone formation. These autoantibodies seem to target intracellular stress proteins, bone remodeling factors, immune regulatory proteins, and microbial antigens [128, 129]. Table 1 illustrates the current understanding of clinically significant autoantibodies in SpA, including their prevalence, mechanisms of action, and clinical use.

**Table 1:** Common autoantibodies in SpA

**Autoantibody**	**Target/Disease**	**Clinical Findings/relevance**	Clinical Utility/Notes	**Evidence Level**	**Hypothesized Microbiota/Mechanistic, Link**	**References**
Anti-CD74	CD74/axSpA	Elevated in axSpA; modest clinical usefulness; no correlation with gut inflammation	Adjunct diagnostic marker; no gut inflammation correlation	2b (Human)	Possible microbial mimicry; gut-driven Th17 activation	[128, 129]
Anti-PsoP27	PsoP27/PsA, RA	Detected in synovial fluid; correlates with CRP in PsA; no correlation with RA activity	Reflects local inflammation; limited disease specificity	2b (Human, synovial fluid)	Th17 polarization in psoriasis; gut–skin–joint axis	[4, 130]
Anti-PPM1A	PPM1A/AS	Elevated in AS, especially advanced sacroiliitis; decreases after TNF inhibitor therapy; correlates with BASDAI	Potential biomarker for disease activity; early-stage diagnostic adjunct	2b (Human)	Inflammation-driven; speculative gut link	[131–134]
Anti-SOST	Sclerostin/axSpA	Elevated in axSpA/IBD; inversely correlated with duration of articular manifestations	Early diagnostic marker in axSpA/IBD; limited replication	2b (Human, limited replication)	IL-17 mediated bone signaling; gut-bone axis	[135–139]
Kaiso/axSpA	Kaiso/axSpA	Elevated in early AS; correlates with CRP and BASDAI; decreases in late-stage nr-axSpA	Early disease biomarker; limited cohort validation	3b (Human, small cohorts)	Possible microbial antigen-driven immune activation	[132, 140–150]
Anti- B2M	Beta-2 microglobulin/AS, SLE, RA, Sjögren’s	Elevated in multiple autoimmune diseases; highest in AS	Reflects chronic immune activation; non-specific	2b(Human, cross-sectional)	Gut-driven systemic immune activation	[151–160]
Anti-HSP65	HSP65/AS	Elevated in some AS patients; small sample sizes; limited statistical significance	Weak evidence; not clinically validated	3b (Human, small observational studies)	Bacterial HSP cross-reactivity	[161, 162]
Anti-14–3-3 eta	14–3-3η/AS, RA	Elevated in AS; correlates with SIJ inflammation, CRP, mSASSS, spine progression	Disease activity marker; requires replication	2b (Human)	Intracellular proteins released during stress trigger immune responses	[163–167]
Anti-MCV	Citrullinated vimentin/AS, PsA, RA	Detected in AS, PsA, RA; higher in AS than controls; associated with higher ESR	Exploratory; not disease-specific	3b (Human, cross-sectional)	Microbiota-induced citrullination (hypothetical)	[127, 162, 168]
Autoantibodies targeting Microbial antigens	ASCA, anti-OmpC, anti-CBir1/AS, IBD	Detected in AS and IBD; prevalence variable	Research biomarker; not disease-specific	2b (Human, serological studies)	Direct gut microbiota dysbiosis	[169–174]
Anti-IL-36Ra	IL-36Ra/PsA, Pso	Detected in 4.72% of PsA and 5% of Pso patients; not detected in most other conditions	Potential pathogenic role; low prevalence	3b (Human, small cohorts)	Dysbiosis-induced Th17/IL-36 signaling	[175]

^*^Evidence Level Definitions:

Level 2b: Individual cohort studies or small clinical studies with moderate consistency

Level 3b: Observational, exploratory findings, or small cohorts without independent replication

Proposed links between gut microbiota and autoantibody production are based on biological plausibility and indirect evidence and should not be interpreted as causally established

### Antibodies against CD74 expressed in axSpA

CD74, a surface glycoprotein expressed on monocytes and macrophages, functions as a receptor for macrophage migration inhibitory factor (MIF), modulating inflammatory responses and osteogenesis. Anti-CD74 antibodies have been observed at higher levels in axSpA patients than in healthy controls or in those with non-specific low back pain. Likelihood ratios suggest a modest level of clinical utility [128].

Despite this, another study found no discernible relationship between anti-CD74 titers and the incidence of gastrointestinal inflammation [129], which may reflect systemic immune activation rather than gut-specific pathogenesis.

Mechanistically, microbial antigens may elicit CD74-mediated responses via molecular mimicry [35]; however, direct evidence in SpA remains limited. Overall, anti-CD74 may serve as an additional biomarker for the early diagnosis of axSpA.

### Anti- PsoP27

PsoP27, a proteolytic product of SERPINB3/B4, is produced by Mast cells in psoriatic lesions and acts as an autoantigen in PsA [176]. Synovial fluid from PsA and RA patients contains antibodies against PsoP27, with higher levels correlating with CRP in PsA but not in RA. Although these antibodies reflect localized joint inflammation, their specificity for PsA is limited [130]. The presence of anti-PsoP27 antibodies may be influenced by microbial-induced Th17 polarization in psoriasis [131], but further research is required to understand gut-mediated mechanisms.

### Anti-PPM1A

Protein Phosphatase Magnesium-Dependent 1A (PPM1A) regulates the BMP and Wnt signaling pathways critical for bone formation and osteoblast differentiation [131, 132]. Anti-PPM1A antibodies are elevated in AS patients, correlating with SIJ severity and responsiveness to TNF inhibitor treatment [132].

Elevated anti-PPM1A titers may indicate dysregulated osteoimmune responses and systemic inflammatory activity [133, 177]. While no direct association with the microbiota has been shown, dysbiosis-induced proinflammatory cytokines may contribute to the loss of tolerance to PPM1A.

### Anti- Sclerostin (Anti-SOST)

Impeded Sclerostin inhibits bone formation by antagonizing Wnt signaling [135, 178]. Reduced Sclerostin levels and elevated anti-SOST levels have been detected in axSpA patients, particularly those with concomitant IBD [139], suggesting a role in the early disease stage. Gut dysbiosis may indirectly influence sclerostin pathways via IL-17–mediated osteoblast activation; however, direct human data are limited [179].

### Anti-Kaiso

Kaiso, a transcriptional regulator belonging to the POZ-zinc finger family, influences Wnt signaling and osteoblast development [180, 181]. Anti-Kaiso antibodies are prevalent in early AS and nr-axSpA, correlating with C-reactive protein and BASDAI scores [140].

These antibodies may arise during initial immunological dysregulation, perhaps associated with microbial antigen exposure at mucosal locations [182, 183], but mechanistic investigations remain in the basic stages.

### Anti-Beta-2 microglobulin (Anti-B2M)

Beta-2 microglobulin (B2M) is an integral component of the MHC class I molecule, essential for antigen presentation. Increased levels of anti-B2M antibodies are observed in AS, SLE, RA, and Sjögren’s disease, with the highest levels in AS patients. These antibodies may indicate persistent immunological activity rather than disease-specific autoimmunity [161]. Systemic inflammation induced by dysbiosis may influence B2M immune responses; however, direct causative evidence in SpA is absent.

### Anti-HSP65

Heat shock protein 65 (HSP65) is elicited in response to cellular stress. Some AS patients have elevated anti-HSP65 levels [161] However, the limited cohort sizes and inconsistent results indicate poor evidence. Cross-reactivity with bacterial HSPs suggests a potential microbial etiology, consistent with the concept of molecular mimicry; yet, the existing human data are insufficient.

### Anti-14-3-3η

14-3-3η is a regulatory protein involved in cellular signaling, released in response to stress, and may provoke autoantibody responses. Higher levels of anti-14-3-3η are found in AS and are associated with SIJ inflammation, CRP, and radiographic spine development [163].

### Anti-MCV

Anti-MCV antibodies Identify Mutated citrullinated Vimentin, a post-translationally modified protein. These antibodies are detected in AS, PsA, and RA patients, with higher levels in AS than in healthy individuals. Anti-MCV correlates with increased Erythrocyte Sedimentation Rate (ESR) but has limited disease specificity [127, 162]. The mechanism of protein citrullination mediated by the microbiota remains conceptual and requires further confirmation [184–186].

### Antibodies targeting microbial antigens

Individuals with AS exhibit antibodies that target gut microbial antigens, such as anti-outer membrane porin C (anti-OmpC), anti-Saccharomyces cerevisiae antibodies (ASCA), and anti-CBir1, which targets flagellin [127]. These interactions reveal systemic exposure to intestinal bacteria and indicate that dysbiosis may stimulate autoimmunity through molecular mimicry or barrier dysfunction [187, 188]. Despite variability in incidence across studies [169, 170], these antibodies may indicate the gut-immune axis rather than as disease-specific biomarkers.

### IL-36Ra

Autoantibodies targeting the IL-36 receptor antagonist (IL-36Ra) have been identified in a small percentage of patients with PsA (4.72%) and Pso (5.0%). These antibodies form immunological complexes, reduce IL-36Ra bioavailability, and may enhance IL-36–mediated inflammation [175]. Although the incidence is minimal, the finding underscores a possible gut-immune-skin-joint axis, especially when microbial dysbiosis enhances Th17-mediated IL-36 signaling.

Generally, the majority of identified autoantibodies in SpA are supported by observational human studies (Level 2b–3b), with limited replication and heterogeneous assay methodologies, highlighting their exploratory rather than definitive diagnostic role.

## Therapeutic modulation of the gut microbiota in SpA

The growing recognition of the gut–immune axis in inflammatory diseases has sparked interest in therapeutic approaches to modify the intestinal microbiota. Experimental and clinical studies suggest that microbial communities may influence immune regulation through mechanisms including modulation of Th17 responses, production of microbial metabolites, and maintenance of intestinal barrier integrity, all of which are linked to the pathogenesis of SpA (Sect. " Mechanisms underlying the gut microbiota-immune axis"). These observations have led to the concept that restoring microbial balance may act as an adjunctive therapy approach in immune-mediated illnesses.

Consequently, several microbiome-targeted therapies have been investigated, including probiotics, dietary prebiotics, microorganisms utilized as medicinal agents, and fecal microbiota transplantation. The strength of evidence varies significantly across techniques, with the majority of mechanistic support originating from preclinical models rather than human clinical studies [34, 90, 189–191].

### Preclinical evidence

Preclinical research has provided important insights into how some bacterial species can impact immunological pathways involved with autoimmune and inflammatory illnesses. Unlike traditional probiotic formulations, which typically include *Lactobacillus, Bifidobacterium,* and *Streptococcus* species [192–196], some experimental studies have investigated the use of bacteria as Drugs (BRUGs), microbial strains selected based on disease-specific changes in the gut microbiome [34, 192, 193].

This technique suggests that restoring bacterial taxa reduced in disease states may provide more targeted immunomodulatory advantages than general probiotic treatment. Consequently, various experimental investigations have explored the therapeutic potential of specific bacterial species [193, 194].

One species that has generated significant attention is *Prevotella histicola*. Studies on EAE, a murine model for MS, demonstrated that colonization with *P. histicola* reduced disease severity and attenuated inflammatory responses [195–197]. In certain experimental contexts, the therapeutic efficacy of *P. histicola* was comparable to that of glatiramer acetate (Copaxone), a commonly utilized disease-modifying therapy for MS [196]. Furthermore, treatment with *P. histicola* reduced arthritis severity in a transgenic HLA-DR4 mouse model of CIA, suggesting that this organism may exert broad immunomodulatory effects across autoimmune disorders [198].

*Bacteroides fragilis* is another well-investigated species, especially those that produce the immunomodulatory chemical polysaccharide A (PSA) [199–202]. Experimental investigations indicate that PSA-producing *B. fragilis* can affect host immunological responses by facilitating Treg differentiation and stimulating IL-10 production. In animal models, these processes have been associated with decreased disease severity in conditions such as colitis and EAE. Mutant strains deficient in PSA did not promote immunological maturation or confer disease protection, underscoring the essential function of this molecule in facilitating the immunomodulatory actions of *B. fragilis* [34, 200, 201, 203, 204].

These preclinical studies collectively indicate that some bacterial taxa may modulate immune regulation by activating Tregs and promoting the generation of anti-inflammatory cytokines. However, it is important to note that the majority of these findings originate from animal models, and their applicability to human SpA has yet to be determined.

### Human clinical studies

Clinical studies examining microbiota-targeted therapies in autoimmune diseases have predominantly focused on probiotic supplements. Probiotics are living bacteria that provide health benefits when consumed in sufficient quantities. Notwithstanding the growing interest in their immunomodulatory capabilities, the therapeutic effectiveness of probiotics in autoimmune disorders remains variable [34].

Multiple clinical investigations on immune-mediated illnesses clarify the potential and limitations of probiotic therapies. Hatakka et al. conducted a randomized study evaluating the impact of *Lactobacillus rhamnosus GG* (LGG) supplementation in patients with RA. Despite probiotic therapy not significantly altering inflammatory markers or clinical indices of illness, patients receiving LGG showed improvements in subjective well-being [205].

A randomized double-blinded placebo-controlled trial involving patients with MS indicated that a combination of probiotics comprising *Lactobacillus casei, Lactobacillus acidophilus,* and *Bifidobacterium bifidum *was linked to an improvement in inflammation-related markers and Expanded Disability Status Scale (EDSS) scores [206]. A research study on the multi-strain probiotic product VSL3 demonstrated changes in gut microbial composition. It increased anti-inflammatory immune responses after 8 weeks of treatment, but did not significantly influence disease progression [207].

Evidence specifically in SpA remains limited. A randomized controlled experiment with 63 individuals diagnosed with AS assessed a probiotic formulation of *Bifidobacterium lactis LAFTI B94, Lactobacillus acidophilus,* and *Streptococcus salivarius*. This research demonstrated that administration of probiotic supplements did not exhibit substantial therapeutic advantages relative to a placebo [208].

These data suggest that while probiotic administration may affect immune responses and microbial composition, consistent clinical effectiveness in SpA and other autoimmune conditions has not yet been established [34], summarized in Table 2.

**Table 2:** Summary of microbiome-driven therapeutic approaches in autoimmune diseases

Therapeutic Strategy	Target condition	Microbial Agent/Intervention	Key Findings	**References**
Probiotic-Based Therapy	AS	*B. lactis LAFTI B94, L. acidophilus, S. salivarius*	No significant clinical benefit vs placebo	[208]
	RA	*LGG*	Improved patient well-being; no significant effect on clinical or inflammatory markers	[205]
	MS	*B. bifidum, L. acidophilus, L. casei*	Improved EDSS scores and inflammatory markers	[206]
	MS	*VSL3 (Streptococcus, Bifidobacterium, Lactobacillus)*	Altered microbiota and induced anti-inflammatory response; no effect on disease progression	[207, 208]
Bacteria as Drugs (BRUGs)	MS	*Prevotella histicola*	Reduced disease severity; comparable to glatiramer acetate (Copaxone) in animal models	[195–197]
	RA	*Prevotella histicola*	Suppressed arthritis in the HLA-DR4 mouse model	[198]
	MS, Colitis	*Bacteroides fragilis (PSA* +*)*	Immunoregulation via Treg induction and IL-10; reduced disease severity	[199–204]
Prebiotics and Dietary Interventions	General Immune Modulation	GOS	Increased IL-10, enhanced NK cell activity	[209, 210]
	Pediatric Immunity Enhancement	FOS	Reduced infection burden; improved vaccine response	[211–214]

### Dietary prebiotics and microbial metabolite modulation

Dietary components are another possible approach to altering the gut microbiota and its metabolic functions. Prebiotics are substrates preferentially used by host microorganisms that confer health benefits by promoting the synthesis of bioactive substances [215]. Common prebiotics include oligosaccharides such as galacto-oligosaccharides (GOS) and fructo-oligosaccharides (FOS), resistant starch, and fructans, which enhance the proliferation of beneficial bacterial communities and encourage the synthesis of SCFAs [215–217]. Moreover, some non-carbohydrate substances, such as cocoa-derived flavanols, have been shown to have prebiotic-like effects by selectively enhancing beneficial microbial populations [215, 218, 219].

Multiple studies have investigated the immunological effects of prebiotic supplementation. For instance, GOS consumption has been linked to elevated IL-10 levels and increased natural killer cell activity in adults [209, 210]. Additional studies have shown that consumption of oligosaccharides may reduce the risk of atopic dermatitis in babies and improve immune responses to viral vaccines [220–223]. Similarly, FOS supplementation has been related to improved antibody responses after influenza vaccination and a lower incidence of febrile illness in pediatric populations [211, 212, 224, 225].

However, it is essential to emphasize that a significant portion of this evidence derives from research examining healthy adults or pediatric populations, rather than those with inflammatory rheumatic illnesses. Thus, the correlation of these data to SpA remains unclear.

## Constraints, adverse outcomes, and safety considerations

Despite growing interest in microbiota-targeted therapy, several obstacles currently hinder its practical implementation in SpA. Initially, data from human clinical studies are few and varied. Numerous studies exhibit small sample sizes, short follow-up durations, and significant diversity in probiotic strains, doses, and treatment lengths, thereby complicating inter-study comparisons [71, 226].

Secondly, several prospective therapeutic microorganisms, such as *Prevotella histicola* and PSA-producing *Bacteroides fragilis*, have shown beneficial effects mostly in animal models. Their safety, effectiveness, and long-term consequences in humans are mostly unexamined [227].

Third, safety issues must be taken into account. While probiotics and prebiotics are often considered harmless, adverse effects have been documented in certain vulnerable populations. Rare problems, including bacteremia, endocarditis, and sepsis, have been reported after probiotic use, especially in patients with significant immunodeficiency, impaired intestinal barrier function, or critical illness [228]. Prebiotics are often well tolerated; however, they may cause gastrointestinal symptoms, including bloating, stomach pain, or osmotic diarrhea, owing to their fermentative properties [215, 228].

Finally, microbiome composition is also influenced by several confounding factors, such as diet, drugs, regional variation, and disease activity, which may limit the repeatability of treatment benefits [229, 230]. These issues underscore the need for meticulously designed longitudinal investigations and controlled clinical trials to determine whether microbiota-targeted therapies may offer significant therapeutic benefits in SpA.

## Methodological limitations and evidence gaps

Microbiome research has significantly enhanced our understanding of the potential role of gut microbial populations in autoimmune diseases, including SpA. Nonetheless, many methodological constraints hinder the interpretation and reproducibility of current findings.

A major drawback is that most research relies on cross-sectional designs and primarily describes microbial composition at a single time point. Although these methods may detect correlations between dysbiosis and illness, they cannot determine causation or elucidate whether the identified microbial changes are a cause or a result of inflammation. Moreover, compositional studies alone provide a limited understanding of microbial activity, leading to an inadequate characterization of mechanistic connections between certain taxa and immunological pathways, such as Th17 activation or Treg regulation (Sect. "Mechanisms underlying the gut microbiota-immune axis").

Technical unpredictability makes the field much more difficult. There is now no globally accepted procedure for microbiome investigations, especially for sample collection, storage conditions, DNA extraction techniques, and sequencing methodologies. Variations in 16S rRNA primer selection and library preparation can introduce significant bias, limiting the comparability of findings and ultimately affecting the reliability of conclusions drawn from microbiome studies [231, 232].

Furthermore, variability in bioinformatic pipelines and statistical methods constitutes an additional obstacle. Disparities in data processing, normalization techniques, and management of numerous comparisons may result in inconsistent outcomes. The conventional use of Operational Taxonomic Units (OTUs) with a 97% sequence similarity criterion has also been increasingly questioned due to insufficient taxonomic precision. This method has been replaced by higher-resolution techniques based on amplicon sequence variant(ASVs), facilitating more accurate and repeatable microbial categorization [233–235].

Another significant factor is the intrinsic inter-individual heterogeneity of the human microbiome. It is expected that only a limited fraction of microbial taxa are shared across individuals within a given community, complicating the identification of disease-specific microbial signatures and limiting generalizability across geographic and demographic categories.

Microbiome datasets are naturally high-dimensional and compositional, necessitating suitable statistical frameworks. Neglecting to account for these characteristics, particularly due to insufficient adjustment for multiple testing, may increase the likelihood of false-positive findings, leading to misleading conclusions about the relationships between microbiome composition and health outcomes.

Despite these constraints, accumulating data support a significant role for the gut microbiota in immune modulation and disease development. Overcoming these methodological obstacles via standardized procedures, longitudinal research designs, and the integration of functional techniques (e.g., metagenomics, metabolomics, and transcriptomics) is critical to advancing the field and enhancing causal inference.

## Future directions and conclusion

Emerging evidence highlights the central role of the gut microbiota in shaping immune responses relevant to SpA and related autoimmune diseases. As discussed in earlier sections, dysbiosis may contribute to disease pathogenesis through multiple mechanisms, including activation of the Th17 axis, molecular mimicry, disruption of intestinal barrier integrity, and altered production of microbial metabolites. These pathways may ultimately promote loss of immune tolerance and the development of autoantibody responses (see Sects. "Mechanisms underlying the gut microbiota- immune axis" and "Autoantibodies in SpA: Clinical Evidence and Mechanistic Links").

Concurrently, host genetic determinants, specifically HLA-B27, interact with the microbiome to further affect disease susceptibility and progression, highlighting the complexity of the gut–immune axis in SpA.

Despite the potential of microbiome-targeted therapies, such as probiotics, BRUGs, and dietary manipulation, as therapeutic options (Sect. " Therapeutic modulation of the gut microbiota in SpA"), existing data are inadequate to endorse their regular clinical use. As emphasized, several findings originate from preclinical models, whereas clinical research has shown inconsistent outcomes.

Consequently, future research should focus on numerous critical domains. Initially, well-designed, longitudinal clinical trials are essential to elucidate the causal links between microbiome modifications and disease activity. Secondly, functional research using multi-omics techniques will be crucial for clarifying the molecular processes linking microbial communities to immunological dysregulation. Third, enhanced standardization of microbiome methodology will increase repeatability and enable cross-study comparisons.

A comprehensive understanding of the interconnections among gut microbiota, host genetics, and immune responses may facilitate the creation of tailored, individualized therapy approaches for SpA. As the topic continues to develop, ongoing multidisciplinary collaboration will be essential for converting microbiome research into therapeutically significant applications.

## Data Availability

No datasets were generated or analysed during the current study.

## Abbreviations

ASCA: Anti-Saccharomyces cerevisiae Antibodies
ASVs: Amplicon Sequence Variants
axSpA: Axial Spondyloarthritis
BASDAI: Bath Ankylosing Spondylitis Disease Activity Index
BMP: Bone Morphogenetic Protein
BRUGs: Bacteria as Drugs
CIA: Collagen-Induced Arthritis
CRP: C-Reactive Protein
DCs: Dendritic Cells
EDSS: Expanded Disability Status Scale
ER: Endoplasmic Reticulum
ERA: Enthesitis-Related Arthritis
ESR: Erythrocyte Sedimentation Rate
FOS: Fructo-Oligosaccharides
GOS: Galacto-Oligosaccharides
HLA-B27: Human Leukocyte Antigen B27
HLA-DR4: Human Leukocyte Antigen DR4
IBD: Inflammatory Bowel Disease
IBS: Irritable Bowel Syndrome
IFN-γ: Interferon Gamma
IgA: Immunoglobulin A
IL-10: Interleukin 10
IL-17: Interleukin 17
IL-22: Interleukin 22
IL-23: Interleukin 23
IL-36: Interleukin 36
IL-36Ra: Interleukin 36 Receptor Antagonist
ILC3: Innate Lymphoid Cells Type 3
K/BxN: K/BxN Mouse Model of Arthritis
LGG: Lactobacillus rhamnosus GG
LPS: Lipopolysaccharide
MAIT: Mucosal-Associated Invariant T Cells
MCV: Mutated Citrullinated Vimentin
MHC: Major Histocompatibility Complex
MIF: Macrophage Migration Inhibitory Factor
MRI: Magnetic Resonance Imaging
NKT: Natural Killer T Cells
NOD2: Nucleotide-Binding Oligomerization Domain Containing 2
nr-axSpA: Non-Radiographic Axial Spondyloarthritis
OTUs: Operational Taxonomic Units
pDCs: Plasmacytoid Dendritic Cells
pSpA: Peripheral Spondyloarthritis
PPM1A: Protein Phosphatase Magnesium-Dependent 1A
Pso: Psoriasis
RA: Rheumatoid Arthritis
ReA: Reactive Arthritis
r-axSpA: Radiographic Axial Spondyloarthritis
SCFAs: Short-Chain Fatty Acids
SFB: Segmented Filamentous Bacteria
SIJ: Sacroiliac Joint
SLE: Systemic Lupus Erythematosus
SpA: Spondyloarthritis
Tfh: T Follicular Helper Cells
Th1: T Helper 1 Cells
Th17: T Helper 17 Cells
TJ: Tight Junction
TNF-α: Tumor Necrosis Factor Alpha
Tregs: Regulatory T Cells
UPR: Unfolded Protein Response
Wnt: Wingless/Integrated Signaling Pathway
anti-B2M: Anti Beta-2 Microglobulin
anti-CBir1: Anti-Flagellin Antibody CBir1
anti-CD74: Anti Cluster of Differentiation 74
anti-HSP65: Anti Heat Shock Protein 65
anti-Kaiso: Anti-Kaiso Protein
anti-MCV: Anti-Mutated Citrullinated Vimentin
anti-OmpC: Anti Outer Membrane Porin C
anti-PPM1A: Anti-PPM1A: Anti-Protein Phosphatase Magnesium-Dependent 1A
anti-PsoP27: Anti Psoriasis-Associated Antigen PsoP27
anti-SOST: Anti-Sclerostin
γδT: Gamma Delta T Cells
APCs: Antigen-Presenting Cells
AS: Ankylosing Spondylitis
EAE: Experimental Autoimmune Encephalomyelitis
MS: Multiple Sclerosis
PSA: Polysaccharide A
PsA: Psoriatic Arthritis
